# Correlation between the Korean Version of the Trunk Control Measurement Scale and the Selective Control Assessment of the Lower Extremity Scores in Children with Cerebral Palsy

**DOI:** 10.3390/medicina57070687

**Published:** 2021-07-06

**Authors:** Misoo Lim, Haneul Lee, Hyoungwon Lim

**Affiliations:** 1 Department of Physical Therapy, Graduate School, Dankook University, Cheonan 31116, Korea; altn6213@naver.com; 2 Department of Physical Therapy, Gachon University, Incheon 21936, Korea; leehaneul84@gachon.ac.kr; 3 Department of Physical Therapy, Dankook University, Cheonan 31116, Korea

**Keywords:** selective control assessment of the lower extremity, trunk control measurement scale, cerebral palsy, evaluation tool

## Abstract

*Background and Objectives*: The purpose of this study was to investigate the correlation between the Korean version of the trunk control measurement scale (K-TCMS) and the selective control assessment of the lower extremity (SCALE). Through this, we tried to find out the effect of proximal stabilization on distal motor development. *Materials and Methods*: Fifty-one children with gross motor function classification system level I–III, diagnosed with cerebral palsy (CP), were studied. The K-TCMS was used to evaluate the body control ability of the children. SCALE was used to quantify selective voluntary motor control (SVMC). *Results*: Analysis of SCALE and K-TCMS showed a significant positive correlation in all items. Multiple regression analysis showed that the SCALE score decreased as age increased, and that it increased as the static sitting balance ability score and the dynamic sitting balance ability score of the K-TCMS increased significantly (*p* < 0.05). *Conclusions*: In children with cerebral palsy, there was a close correlation between trunk control and selective voluntary motor control of the lower extremities. Therefore, when trying to improve the lower extremity function of a child with cerebral palsy, a trunk control intervention should be considered.

## 1. Introduction

Cerebral palsy (CP), which is due to a non-progressive disorder occurring in the brain, is a permanent impairment of movement and postural development, which causes activity limitation [1]. With the maturation and development of the CNS at about 18 weeks of age, the sagittal stabilization of the trunk occurs, which sequentially establishes movements occurring in the transverse plane, such as rolling, creeping, and crawling [2]. Insufficient trunk control and postural adjustments can cause difficulties in performing these functions in children with cerebral palsy [2]. The ability of children with CP to regulate the trunk is the basis for planning interventions to improve activity and participation [3].

Selective voluntary motor control (SVMC) is defined as the ability to activate and separate muscles based on voluntary exercise or posture needs [4]. The lateral corticospinal tract (CST) regulates both directionality and the force generation involved, creating voluntary movement. Damage to the lateral CST interferes with the strength, speed, and timing of voluntary movement patterns [5]. Previous studies revealed that lateral CST damage in the periventricular white matter (PWM) is associated with motor impairment in CP. The absence of SVMC can result in a synergy pattern that disappears over time in infants without brain lesions. However, it remains in premature babies, who could suffer damage to the white matter area [6].

Heyrman et al. developed the trunk control measurement scale (TCMS), a measure of trunk control in CP [7], and Ko and Jung developed the K-TCMS, which is suitable for the Korean population [8]. The TCMS is used to determine the link between trunk control and functional activity in CP and to plan interventions. Recently, the TCMS has been used to screen children in order to investigate the relationship between trunk control ability and gait in patients with CP who can walk [9].

SVMC disorders in CP cause a vicious cycle of movement limitations, joint contracture, motor dysfunction, and decreased activity [10,11]. SCALE (selective control assessment of the lower extremity) can assess selective voluntary motor control of the lower limbs in CP, and the scores are clearly and easily identified [6,12].

Measurement tools must be sensitive enough to detect clinically relevant changes as the greater the sensitivity of the assessment, the easier it is to detect any improvement after intervention, or deterioration over time [13]. In pediatric physical therapy, most of these assessment tools are used to gauge functional movement, and there is a lack of tools that can directly assess movement disorders in children with CP. In addition, an objective examination of the relationship with the tools developed in the past can provide a sufficient understanding of functional impairment [14]. Therefore, it is necessary to analyze the correlations between reliable evaluation tools to obtain a better understanding of functional impairment [14].

Human motor development is achieved by the functional use of the trunk and limb muscles [15]. Gross motor skills mainly require the use of proximal and axial muscles for posture control and locomotion [16,17,18,19,20], while fine motor skills require more precise movements, such as the functional use of the hands [21,22,23].

Gross motor skills are required for control of the proximal muscles for locomotion or postural control [16,17,18,19,20]. In a previous study, the relationships between motor function and trunk control [9], trunk control and balance [24], and SCALE and PBS in children with CP [14] were investigated, and, as a result, a high correlation was reported. Therefore, it can be seen that trunk control has an effect on the motor control of the distal part and has a close correlation.

On the other hand, other studies investigated the developmental relationship between postural stability and limb muscles, and reported unclear (negative) results [25].

However, in recent clinical trials, it has been reported that trunk control intervention based on neurodevelopmental treatment improves postural control, balance, and gross motor functions in children with CP more effectively than conventional treatments [26,27,28].

With this as background, we have set up two hypotheses: (1) there will be a significant (high) correlation between TCMS and SCALE; and (2) the development of postural stability in the proximal part of the body will precede the development of distal motor function.

There is no consensus on the correlation between trunk control and lower extremity motor functioning. Therefore, in this study, we aimed to investigate the correlation between TCMS and SCALE in children with CP and tried to find out whether stabilization of the proximal part precedes the development of distal motor neuron function.

## 2. Materials and Methods

### 2.1. Ethical Approval

This study was approved by the Dankook University Institutional Review Board (approval no. 2019-05-22 and approval date 2019-06-11). All participants gave their written informed consent prior to participation in the study. This observational study employs the “strengthening the reporting of observational studies in epidemiology” (STROBE) procedure.

### 2.2. Participants

The children of this study were being treated in physical therapy rooms in various metropolitan cities and regions in Korea, with participation from 51 children diagnosed with spastic cerebral palsy. The subjects were children of the gross motor function classification system (GMFCS) level I–III, who were from 4 to 15 years old and could understand and follow the therapist’s instructions. This study excluded children who had received botulinum toxin A or underwent orthopedic surgery within the last 6 months. The researcher explained the purpose and procedure of the study to the child and the parents and asked for consent in order to evaluate the subject’s trunk control ability and selective exercise control ability. The evaluation was conducted on those children who agreed to participate in the study.

### 2.3. Measurement Tools

#### 2.3.1. Korean Version of Trunk Control Measurement Scale: K-TCMS

K-TCMS consists of 15 items. The subject is asked to sit on the table to perform a functional activity, and the subject’s balance in the sitting position, that is, the ability to control the trunk, is evaluated. The body control measurement scale consists of three subscales: static sitting balance, dynamic sitting balance, and dynamic reaching or equilibrium reaction [8]. In the static sitting balance, the static adjustment of the trunk during the upper and lower extremity exercise is evaluated. Dynamic sitting balance evaluates the active movement of the trunk within the base during movement [7]. The general starting position for each item (sitting across the edge of the table without supporting the arms or feet) must be observed. In “static sitting balance”, 5 items are evaluated and scored within the range 0–20. In “dynamic sitting balance”, 7 broad parameters are evaluated. Each parameter consists of detailed items, a total of 16 items are evaluated, and the range of total points is 0–28. The “dynamic reaching or equilibrium response” evaluates a total of 3 items and has a score range of 0–10. A total of 35 items are evaluated, including those that measure left and right separately. The total score ranges from 0 to 58 points [8].

The reliability of the K-TCMS between testers was more than ICC 0.9, which was similar to the ICC = 0.94–0.98. The test-retest reliability value was ICC = 0.902–0.962, which was similar to the original test-retest reliability value [7,8].

#### 2.3.2. Selective Control Assessment of the Lower Extremity: SCALE

SCALE can be measured without special tools and consists of a 3-point scale from 0 to 2 points. The hip joint evaluation is performed while lying on one side, and the rest of the evaluation is performed in the sitting position. The evaluator assists with the weight of the limbs, but not with the exercise. For each joint, points are given as “normal” (2 points), “impaired” (1 point), and “unable” (0 points). If the desired sequence of motion is completed within three seconds without movement of the ipsilateral or contralateral distal joint that is not required by the subject, a “normal” grade is awarded. If the subject isolates the motion during the measurement but exhibits errors such as one-way motion, less than 50% of the available range of motions, undesired joint motion (including mirror motion), and takes more than a three-second verbal count, an “impaired” grade is awarded. An “unable” grade is awarded when the subject does not initiate the required exercise or when the subject shows a synergistic mass flexor or extensor pattern [5,14]. A total of 5 joints are evaluated for the left and right sides, each joint is scored between 0–4 points by summing both sides, and the range of total points is 0–20. SCALE showed high reliability, with an ICC of 0.88–0.91 in the test, and showed a significant inverse correlation with Spearman’s rank correlation coefficient *p* = 0.83 (*p* < 0.001) in comparison with GMFCS for validity [5].

### 2.4. Procedure

SCALE was used to evaluate the selective motor control ability of children with cerebral palsy, and K-TCMS was used to evaluate the body control ability. The evaluation was assessed and scored by a therapist with 5 years of experience in pediatric physiotherapy. The order of measurement of K-TCMS and SCALE was randomized. A 5-min break was given between evaluations, and the highest score among 3 evaluations was selected for each item.

### 2.5. Statistical Analysis

The sample size was estimated by using G*Power 3.1.9. (Heinrich Heine University, Dusseldorf, Germany) [29]. A moderate expected effect f^2^ of 0.20 [30] was ascertained for linear multiple regression, with a level of significance of 0.05 and a power of 0.8. A sample size of 52 was required to show statistical significance when clinically significant differences were at 80.4% power.

All data were analyzed using the statistical program SPSS for Windows (IBM, Armonk, NY, USA). One-way ANOVA and chi-square tests were performed to determine the general characteristics of the children. Pearson’s correlation analysis was performed to examine the correlation between SCALE scores and total scores for each joint, and the scores for each item of the K-TCMS. A stepwise multiple regression analysis was performed to determine the factors that influence the SCALE score. The GMFCS level for regression analysis was substituted with a variable. The level of significance was set at α = 0.05.

## 3. Results

### 3.1. General Characteristics of the Children

The general characteristics of the children are shown in Table 1.

### 3.2. Correlation between Each Joint Score and Total Score for SCALE and Item Score and Total Score of K-TCMS

Table 2 shows the results of the correlation analysis between SCALE and K-TCMS. The correlation between the SCALE score and the total score for each joint, and the item score and total score for each item of K-TCMS, was found to have a significant positive correlation for all items (*p* < 0.01).

### 3.3. Stepwise Multiple Regression Analysis Results for Age, GMFCS Level and K-TCMS Scores Affecting SCALE Score

Table 3 shows the results of the stepwise regression model for age, GMFCS level, and K-TCMS score that affect the SCALE score. In model 1, the most influential variable for the total score from SCALE was static sitting, which increased by 0.824 points for every 1-point increase in static sitting and was statistically significant (*p* < 0.001). In model 2, the most influential variable for the total score from SCALE was static sitting, which increased by 0.835 for every 1-point increase. The next most influential variable was age. The score decreased by 0.271 points as age increased by 1 year and was statistically significant (*p* < 0.001). In model 3, the variable affecting the total score the most from SCALE was static sitting, which increased by 0.579 for every 1-point increase. The next most influential variable was age, and the score decreased by 0.370 each time the age increased by 1 year. The next influential variable was dynamic sitting, which increased by 0.253 points with every 1-point increase and was statistically significant (*p* < 0.001).

The explanatory power of the independent variables, including the variables excluded from each model, for the total score from SCALE was 57.2% in model 1, 62.4% in model 2, and 65.8% in model 3.

## 4. Discussion

Understanding the effects of motor impairment is the basis for planning effective interventions in functional performance. For this, the use of valid and reliable evaluation tools must be given precedence [31]. In this study, we investigated the correlation between TCMS and SCALE in children with CP and tried to find out the effect of proximal stabilization on distal motor development through this correlation.

In this study, the SCALE score and total score of each joint, and the score and total score of each item of the K-TCMS system, exhibited a high correlation. Panibatla et al. reported that impairment of trunk control affects functional abilities, and trunk control and balance ability suggest that it is an essential element of functional ability in CP [24]. Their research suggested that the ability to perform functional activities is affected by the stability of the trunk during movements of the upper and lower extremities. In addition, it was shown that a trunk-targeted intervention to improve the TCMS score increases gross motor function and PBS performance [24]. They also reported a significant correlation between the dynamic component of the PBS and the static component of the TCMS. Previous research also reported a high correlation between SCALE assessments, which can assess the degree of motor impairment in the lower extremities, and the PBS, which is used for the evaluation of functional balance. The researcher suggested that a higher SCALE score improved PBS by increasing the motor function and reducing the synergy pattern [8,14]. As a consequence, the total SCALE score was very useful in explaining the overall functional capacity of the subjects [8,14]. Another previous study reported a strong association between TCMS and GMFCS levels, which supports the previous hypothesis that trunk control is essential for selective movements of the limb and functional capacity [9,32]. Therefore, it can be stated that SVMC of the lower limbs is based on trunk control, which supports the results of this study in terms of the strong correlations between the K-TCMS and the SCALE scores.

In this study, the factors influencing the SCALE score included age and each item of the K-TCMS. Hanna et al. reported that children and adolescents with GMFCS at levels III, IV, and V are more likely to lose motor function [33]. This is because physical growth and a reduction in spontaneous motor function are related to muscle buildup and changes in muscle tone and spine alignment [4,33]. In a previous study, the maximum contractile force of the main lower extremity muscles in dorsiflexion and plantar flexion of spastic CP was shown to be 52% or less compared to that of normal children of the same age [34]. Fowler and Goldberg suggested that they were unable to independently move the hip, knee and ankle joints at various angles, relying on patterns of flexion and extension that were closely coupled to each other [5]. Other studies have also shown that when SVMC is impaired, there is a lack of normal inter-joint coordination, resulting in synergy or coupled movement during the swing phase of the gait [5,35]. In muscles with contracture, the sarcomere, a functional unit of muscle contraction, is almost twice the normal length [36]. Researchers have shown that extremely lengthy muscle fiber segments can result in relatively low active forces [6,36]. Therefore, in SCALE, as the child matures, the muscle hypertonicity due to spasticity is fixed, causing joint contracture and low activity. For this reason, it could be stated that the distal joint is more impaired. Reports from these previous studies can explain the negative correlation, with the lower SCALE scores as the age of patients in this study increased [35,36].

In addition, in this study, the items of static sitting balance and dynamic sitting balance of the K-TCMS showed a positive correlation with the SCALE score. This is consistent with previous studies by Balzer et al., who reported a strong correlation between SCALE and total TCMS scores.

The pyramidal pathway is known to be mainly involved in fine motor functions such as hand function, while the corticoreticular pathway, one of the extrapyramidal pathways, is known to be involved in gross motor functions such as posture control and locomotion [37,38,39,40,41,42,43,44]. This corticoreticular tract status can be used to evaluate the gait function and trunk stability of pediatric patients.

Sasaki et al. investigated whether the neurons supplying the corticospinal neurons that supply the trunk muscles are controlled during the contraction of hands, legs, and jaw muscles [45]. Voluntary contraction of the hand muscles promoted the amplitude of the motor-evoked potential of the trunk muscles. However, voluntary contraction of the leg and jaw muscles did not promote the locomotor amplitude of the trunk muscles [45]. They reported that the spinal neurons that control the trunk muscles are located between the upper and lower extremity muscles, so that the trunk muscles are likely to affect the neurons supplying the upper and lower extremity muscles in the spinal circuit, and prompt each other [45]. This supports the positive correlation between the K-TCMS and the SCALE scores shown in the present study.

On the other hand, previous studies that explored the relationship between postural control and fine motor control produced unclear results. The neural systems responsible for postural control are separate from the neural substrates that underpin control of the hand. Firstly, the nervous system connections responsible for postural control and hand control are separate, and they have low explanatory power by collecting data from a small population and relying on subjective measurements. Next, posture control can be less related to the task specificity of fine motor function or gross motor function. Variations in posture control during standing, and bare-handed control and sitting posture, may be explained because the requirements are different.

Performing trunk rotation during arm-reaching made posture control more difficult in children with CP, and increased trunk movement during walking was associated with decreased performance in sitting, with TCMS [46,47]. They suggested that trunk control affects the gait function of children with CP [48].

In addition, the results of this study are consistent with clinical interventions reporting that a trunk protocol focused on dynamic co-activation of trunk flexors and extensors (based on neurodevelopmental therapy) improved gross motor function in children with spastic cerebral palsy [26,27,28]. Similarly, the same study results were reported in adult stroke patients [49].

Although the multiple regression model was limited by the multicollinearity of some variables, the SCALE score increased as the static and dynamic balance ability scores of K-TCMS increased, showing a close correlation. The dynamic reaching of the K-TCMS showed a significant strong correlation with SCALE. However, this was classified as a result-exclusion variable for the stepwise regression model. This might be because the dynamic reaching item maintains the balance of the trunk on the base of support and performs the work of extending the arm.

In summary, it was reported that a high correlation between trunk control and balance and an improvement in SCALE score improved the PBS by increasing the gross motor function and reducing the synergy pattern. In addition, we confirmed that there is a close correlation between trunk control and selective motor control of the lower extremities, and based on these results, we propose that trunk control is necessary for a stable base of support required to perform functional activities for limb movement [50]. It is meaningful as a study, in that it explains that the stabilization of the proximal part is closely related to the growth of distal motor development. Therefore, the results of this study can be said to support the research hypothesis that the development of postural stability in the proximal part of the body precedes the development of peripheral motor functions.

The limitations of this study are as follows. The children included in this study were only children with GMFCS levels I–III who could sit independently for 30 min or more, and those who were not able to sit independently were not examined. In addition, the results of this study were not generalizable to all children with cerebral palsy because we only included children with spastic CP. Finally, the correlation cannot be proven to depict a causal relationship. In future work, clinical studies will be needed to verify that the application of trunk posture control training to children with CP affects the distal part and has a close correlation.

In conclusion, this study analyzed the correlation between the measurement of trunk control and the evaluation of selective control of the lower extremities in spastic CP. There was a significant positive correlation among all items of the SCALE score for each joint, total score, and the K-TCMS for each item score and total score. This suggests that trunk control is essential for selective movement and functional ability of the lower limbs, and SVMC of the lower limbs is based on the stability of the trunk. From the results of previous studies and the results of this study, it can be estimated that trunk control can affect the selective movement of the lower extremities, and that motor control precedes the selective movement of the lower extremities. Therefore, when trying to improve the lower extremity function of a child with CP, a trunk control intervention should be considered.

## Figures and Tables

**Table 1:** General characteristics of the children who participated.

Characteristics	GMFCS			*p*-Value
	Level Ⅰ (*n* = 23)	Level Ⅱ (*n* = 15)	Level Ⅲ (*n* = 13)	
Age, year (M ± SD)	8.13 ± 3.25	8.33 ± 4.13	10.00 ± 4.22	0.399
Gender				
Female, *n* (%)	14 (60.9)	6 (40.0)	5 (38.5)	0.307
Type				
Diplegia/*n* (%)	6 (26.1)	15 (100.0)	13 (100.0)	< 0.001
Rt. Hemiplegia/*n* (%)	8 (34.8)	-	-	
Lt. Hemiplegia/*n* (%)	9 (39.1)	-	-	

**Table 2:** Correlation between each joint score and total score for SCALE and item score and total score of K-TCMS.

	SCALE_ Hip	SCALE_ Knee	SCALE_ Ankle	SCALE_ Subtalar	SCALE_ Toe	Static Sitting	Dynamic Sitting	Dynamic Reaching	K-TCMS_ TS
SCALE_TS	0.697 **	0.786 **	0.883 **	0.882 **	0.837 **	0.762 **	0.622 **	0.691 **	0.741 **
SCALE_ Hip		0.532 **	0.530 **	0.496 **	0.398 *	0.541 **	0.518 **	0.538 **	0.574 **
SCALE_ Knee			0.612 **	0.573 **	0.566 **	0.796 **	0.590 **	0.693 **	0.737 **
SCALE_ Ankle				0.776 **	0.673 **	0.614 **	0.578 **	0.613 **	0.649 **
SCALE_ Subtalar					0.716 **	0.589 **	0.486 **	0.581 **	0.588 **
SCALE_ Toe						0.605 **	0.408 *	0.445 **	0.522 **
Static sitting							0.739 **	0.831 **	0.913 **
Dynamic sitting								0.777 **	0.936 **
Dynamic reaching									0.914 **

Abbreviations: SCALE, selective control assessment of the lower extremity; K-TCMS, the Korean version of the trunk control measurement scale; TS, total score. * *p* < 0.05. ** *p* < 0.01.

**Table 3:** Stepwise multiple regression analysis results for age, GMFCS level, and the K-TCMS scores affecting the SCALE score.

Model	Independent Variables	B *	S.E	β ^†^	*P*
1	Constant	−0.995	1.707		0.043
	TCMS_static sitting	0.824	0.100	0.762	<0.001
2	constant	1.173	1.780		0.513
	TCMS_static sitting	0.835	0.094	0.772	<0.001
	age	−0.271	0.098	−0.241	0.008
3	constant	2.246	1.752		0.206
	TCMS_static sitting	0.579	0.138	0.536	<0.001
	age	−0.370	0.101	−0.329	<0.001
	TCMS_dynamic sitting	0.253	0.104	0.325	0.019

* Unstandardized coefficient beta, ^†^ standardized coefficients beta. Abbreviations: GMFCS, gross motor function classification system; SCALE, selective control assessment of the lower extremity; K-TCMS, the Korean version of the trunk control measurement scale. Dependent variable: total SCALE score. Except variables; Model 1: age, GMFCS level II, GMFCS level III, TCMS_dynamic sitting, TCMS_dynamic reaching. Model 2: GMFCS level II, GMFCS level III, TCMS_dynamic sitting, TCMS_ dynamic reaching. Model 3: GMFCS level II, GMFCS level III, and TCMS_dynamic reaching. Adjusted R^2^; Model 1: 57.2%, Model 2: 62.4%, Model 3: 65.8%.

## Data Availability

The datasets generated during this study are available from the corresponding author on reasonable request.
